# Optimization of Burgers creep damage model of frozen silty clay based on fuzzy random particle swarm algorithm

**DOI:** 10.1038/s41598-021-98374-1

**Published:** 2021-09-23

**Authors:** Yafeng Yao, Hua Cheng, Jian Lin, Jingchen Ji

**Affiliations:** 1 School of Civil Engineering, Nantong Vocational University, Nantong, 226001 China; 2 Post-Doctoral Research Station of Safety Science and Engineering, Anhui University of Science and Technology, Huainan, 232001 China; 3 Department of Civil Engineering, Anhui Jianzhu University, Hefei, 230601 China

**Keywords:** Environmental sciences, Engineering, Mathematics and computing

## Abstract

The creep characteristics of frozen rock and soil are crucial for construction safety in cases of underground freezing. Uniaxial compression tests and uniaxial creep tests were performed at temperatures of − 10, − 15, − 20, and − 25 °C for silty clay used in Nantong metro freezing construction to investigate the effect law of the stress–strain curves and creep curves. However, owing to the complex effects of factors such as temperature and ground pressure, the mechanical properties of underground frozen silty clay are uncertain. The Burgers creep damage model was established by using an elastic damage element to simulate the accelerated creep stage. The traditional particle swarm optimization algorithm was improved using the inertia weight and the fuzzy random coefficient. The creep parameters of the Burgers damage model were optimized using the improved fuzzy random particle swarm algorithm at different temperatures and pressure levels. Engineering examples indicated that the optimized creep model can more effectively characterize the creep stages of frozen silty clay in Nantong metro freezing construction. The improved fuzzy random particle swarm algorithm has wider engineering applicability and faster convergence than the traditional algorithm.

## Introduction

With the substantial development of the Chinese economy and continuous urban expansion, the urban subway has been rapidly promoted and applied to resolve the shortages of land and environmental resources. To keep pace with the regional economic integration in the Yangtze river delta of China, Nantong (the core city) is constructing metro lines 1 and 2. According to an analysis of the engineering geology, the Nantong region has the characteristics of a soft soil layer. The low strength and poor stability of the soft soil will lead to damage to the foundation and ground settlement. For the Nantong subway tunnel in the soft soil layer, connection channel construction usually adopts the method of shaft excavation or hole excavation, to reduce the disturbance to the stratum caused by excavation. Regardless of which method is adopted, the soil must be strengthened before excavation in the construction of weak strata^1–3^. However, when there is flowing sand in the reinforcement range, the ordinary reinforcement method is difficult to implement. Therefore, the artificial freezing method is often used in this type of engineering. In freezing construction, the frozen rock or soil creeps over time after the tunnel excavation, affecting reliability of the underground structure^4–6^. Scientifically establishing the creep model of frozen soil and accurately describing its deformation characteristics via artificial frozen soil tests are important for the analysis of the tunnel stability and are among the main challenges in frozen soil mechanics.

Many researchers have conducted extensive investigations of the creep model and obtained preliminary results. According to Yang^7^, the creep characteristic curves of warm ice-rich frozen soils at different temperatures were investigated. Based on this, a statistical damage model was established and the model parameters were identified based on the experimental results. Zhang et al.^8^ obtained creep laws under different shear stress levels through in situ shear creep tests of rock and established a shear creep model through the optimized back analysis method. Qi et al.^9^ derived the three-dimensional creep constitutive equation of rock under a constant stress, measured the curve for the whole process of the rheological test of sandstone in the Three Gorges Reservoir Region with a rheological model, and obtained the model parameters. Yuan^10^ developed a parabolic viscoelastic-plastic creep constitutive model for a high stress, wrote finite-element code for the displacement, and then performed a displacement inverse analysis for the excavation process of a frozen shaft with field measurement data to obtain the constitutive mechanical model parameters of frozen soil. By optimizing damage factors, Wang et al.^11^ made full use of the inverse function solution of the sigmoid function in mathematical methods to obtain a new model that can reflect the uniaxial compression creep characteristics of rocks. The practicability of the model was verified through a creep test. By replacing the viscoelastic and viscoplastic parts of the Nishihara model with the fractional derivative Abel dashpot, Sun et al.^12^ derived a new creep constitutive relation. Compared with the traditional Nishihara model, this model can describe not only the decayed and stable creep under low stress level, but also the accelerated creep under high stress levels by adding only one new parameter. According to the elastic theory of porous materials, He et al.^13^ developed a statistical constitutive damage model to investigate the rock damage characteristics under water pressure. Li et al.^14^ carried out Laplace transform on the basis of the Nishihara model, established the fractional derivative creep constitutive model of artificial frozen soil, and deduced the flexible parameter matrix of the model by Newton iteration method. After a large number of creep tests, Wei et al.^15^ analyzed the creep characteristics of columnar jointed basalt rock mass. Then the least square method and simulated annealing algorithm were used to compare the inversion efficiency of creep model parameters. In view of the deficiency that the traditional creep model could not accurately describe the accelerated creep stage, Liu et al.^16^ established an improved Nishihara model of the rock creep by replacing the model parameters with nonlinear ones. On the basis of Burgers model, Li et al. added the aging factor and established the three-dimensional creep damage constitutive model by Laplace transform. Finally, L-M algorithm was used to identify the creep parameters. Shi et al.^17^, Wang et al.^18^ added hardening factor and damage factor to the traditional creep model, and established a new creep damage model based on the test results under different stress conditions. Xu et al.^19^ obtained the creep equation of the visco-plastic model by using the elastic spring and the plastic Hooke-Brown element in series. The analytical solution and closed-form curve of creep deformation of surrounding rock in deep circular tunnel were obtained by experimental analysis. Aiming at the settlement problem in engineering practice, Zhang et al.^20^ proposed a hybrid agent intelligent model for predicting creep coefficient Cα. The combined model introduces particle swarm algorithm in random forest, which overcomes the problems of user experience dependence and local optimization.

To sum up, previous studies on the optimization of frozen soil creep model mostly added damage elements on the basis of traditional creep constitutive relationship and established a new creep model through Laplace transform. According to the test results, the model parameters were obtained to satisfy the creep characteristics under different stress and temperature conditions. In view of the uncertainty of creep characteristics of deep frozen soil in underground engineering, some scholars have adopted conventional intelligent algorithms such as least square method, ant colony algorithm, Bayesian analysis method and particle swarm optimization algorithm to reduce the testing workload in the optimization process of creep parameters and models. Among them, particle swarm optimization algorithm is more suitable for multi-objective parameter optimization of exponential function such as frozen soil creep model equation due to its simple implementation and strong expansibility. However, previous studies show that the optimization efficiency of particle swarm algorithm is generally not high, and the randomness and fuzziness of creep of frozen soil are not taken into account simultaneously. It also causes the failure of the model, which cannot accurately describe the creep characteristics of frozen soil in deep underground engineering, and even causes safety accidents.

Therefore, in this study, combined with the uncertainty distribution of underground frozen soil mechanics, the original particle swarm optimization algorithm was improved by fuzzy random method. Then the new algorithm was used to optimize creep parameters, so as to obtain an effective model to describe the creep characteristics of deep underground frozen soil, and provided basic documents for underground freezing projects in Nantong and the surrounding cities.

## Experiment method and results

### Specimen collection and production

Nantong metro line 1 has a total length of 39.15 km with 25 stations (including 34.75 km underground). To ensure the representability of the test results, undisturbed soils were collected from a typical soil layer of Nantong metro constructed via the freezing method. The silty clay was buried to a depth of 15.42–23.39 m, with a moisture content of 25.57%, a dry density of 2.16 g/cm3 and a saturation degree of 87.3%.

The collected soil samples were carefully sealed in a double-layer plastic preservation package, which was marked and tied with a string. The bound soil sample was packed into the sampling tube, labeled, sealed with tape, and transported to the laboratory safely after packing. The laboratory specimens were produced in strict accordance with the relevant standards (MT/T593-2011) of the Chinese test code for the physical and mechanical properties of artificial frozen soil^21–23^. The package of undisturbed soil was carefully opened, the layer of the soil sample was identified, and the soil sample was sawed flat at both ends using a hacksaw. The soil samples were formed into 50 mm × 100 mm specimens. The minimum size of the samples had to be > 10 times the maximum particle size of the soil samples. The external dimension error was < 1.0%, and the parallelism of the two end faces of the sample was not greater than 0.5 mm.

### Uniaxial compression experiment method

Uniaxial unconfined compressive strength tests at temperatures of − 10, − 15, − 20, and − 25 °C were performed in accordance with MT/T593-2011. First, the pressure was set as 0, the prepared silty clay specimens were arranged coaxially between the top and bottom stress chucks of the test system, and the pressure and deformation test instruments were installed. Then, the pressure gauge was actuated, and the deformation and force were measured simultaneously. When the strain was ≤ 3%, every increase of 0.3%-0.5% was recorded; otherwise, every increase of 0.6%-1.0% was recorded. When the pressure reached the peak value or became stable, the test was stopped by adding a strain of 3%-5%. Finally, the test specimens were removed after unloading, and the characteristics of the damaged sample were examined^24–26^.

### Uniaxial creep experiment method

Before the creep test, the specimen was placed between the top and bottom pressure heads of the creep apparatus, and the specimen surface was sealed to prevent changes in the water content. The dynamometer and displacement meter were well installed and connected. Then, the loading system was started, and the specimen was quickly loaded to the required stress level. During the test, the specimen was subjected to a constant stress, and the time and strain values of the whole process were recorded. When the specimens reached stable deformation $\left( {\frac{d\varepsilon }{{dt}} \le 0.0005\,{\text{h}}^{ - 1} } \right)$ or the deformation rate approached a constant $\left( {\left| {\frac{{d\varepsilon^{2} }}{{dt^{2} }}} \right| \le 0.0005\,{\text{h}}^{ - 2} } \right)$^27,28^, the creep tests were stopped.

### Uniaxial test results

As indicated by the creep curves (Supplementary Appendix), at the same temperature, the creep value increased with increasing stress level. However, the creep value has obvious uncertainty with the change of temperature at the same stress level. In addition, the curves indicated that the creep law was different at different stress levels. When the stress was low or moderate, the creep curve was relatively gentle, whereas when the stress was high, the creep curve exhibited an accelerating trend.

Therefore, in the case of a changeable and complex underground geotechnical environment, to characterize the creep law of frozen soil accurately, it is necessary to employ an intelligent algorithm to conduct fuzzy improvement and obtain accurate parameters to optimize the creep model.

In actual underground freezing engineering, the creep parameters and models have various uncertainties. To avoid the limitations of small-specimen tests and perform parameter inversion and model identification effectively, a fuzzy random analysis method based on intelligent calculations was adopted in this study.

## Methodology

### Traditional particle swarm algorithm

The particle swarm algorithm is based on swarm intelligence and is inspired by life science and iteration theory^29,30^. Professors Kennedy and Eberhart from the United States developed an intelligent algorithm to represent animals foraging effectively via clustering, which can be described mathematically as follows.

Assume that there is a population $x=({x}_{1},{x}_{2},\cdots ,{x}_{n}{)}^{T}$ of *m* particles in an *n*-dimensional search space, where the position of particle *i* (*i* ≤ *m*) can be expressed as ${x}_{i}=({x}_{i,1},{x}_{i,2},\cdots ,{x}_{i,n}{)}^{T}$, and its velocity is ${v}_{i}=({v}_{i,1},{v}_{i,2},\cdots ,{v}_{i,n}{)}^{T}$. According to the initialization process of the algorithm, the position and velocity of each particle are set randomly. After each iteration, the adaptive value of the particle is re-evaluated according to the objective function (evaluation function), and the current adaptive value of each particle is compared with the best adaptive value in the flight path. Then, the local optimal value of the particle is recorded (${p}_{i}=({p}_{i,1},{p}_{i,2},\cdots ,{p}_{i,n}{)}^{T}$). Finally, the current adaptive values of all the particles in the population and all past optimal adaptive values are compared to determine the current global optimal value of the population (${p}_{g}=({p}_{g,1},{p}_{g,2},\cdots ,{p}_{g,n}{)}^{T}$). During the algorithm iteration process, once each particle finds the two aforementioned extreme values^31–33^, it updates its speed and position according to the following two Equations:

1 $$v_{i,d}^{k + 1} = v_{i,d}^{k} + c_{1} rand()\left( {p_{i,d}^{k} - x_{i,d}^{k} } \right) + c_{2} rand()\left( {p_{g,d}^{k} - x_{i,d}^{k} } \right)$$

2 $$x_{i,d}^{k + 1} = x_{i,d}^{k} + v_{i,d}^{k + 1}$$

Here, c_1_ and c_2_ are acceleration constants; rand () is a random function with a value between 0 and 1; ${v}_{i,d}^{k}$ and ${x}_{i,d}^{k}$ represent the velocity and position, respectively, of particle *i* in dimension *d* after *k* iterations; ${p}_{i,d}^{k}$ represents the position of the individual optimal value of particle *i* in dimension *d*; and ${p}_{g,d}^{k}$ represents the position of the global optimal value of the group in dimension *d*. According to this process, the algorithm is iterated until the maximum number of iterations is reached or the error is controlled within the specified range. The iterative algorithm is terminated to achieve the global optimum.

### Fuzzy random improvement

The traditional particle swarm algorithm has few parameters, making it easy to implement for a wide range of applications. In practice, in the process of solving complex high-dimensional problems, the flight direction of particles approaching the optimal region is uncertain, resulting in a relatively low convergence speed of the algorithm. Additionally, although the algorithm is based on random iteration, it cannot accurately reflect the fuzziness of deep underground geotechnical mechanics. Therefore, to improve the applicability and convergence ability of particle swarm optimization, fuzzy random improvement was applied to the original algorithm:

1. In the traditional particle swarm algorithm, if the values of c_1_ and c_2_ are small, the algorithm efficiency will be improved. However, larger values of c_1_ and c_2_ can easily jump out of a local optimality and search for the global optimality. To balance the efficiency and convergence, the inertia weight $\tilde{\omega }$ was introduced to modify the particle velocity Equation.

3 $$v_{i,d}^{k + 1} = \tilde{\omega }v_{i,d}^{k} + c_{1} rand()\left( {p_{i,d}^{k} - x_{i,d}^{k} } \right) + c_{2} rand()\left( {p_{g,d}^{k} - x_{i,d}^{k} } \right)$$

A simulation revealed that the algorithm efficiency and convergence speed can be well balanced when $\stackrel{\sim }{\omega }$ is fuzzy random controlled in the range of [0.4, 0.9]. Considering the fuzziness of the balance, the intermediate fuzzy random distribution function was adopted. According to the fuzzy interval operation, the triangular membership function was used to describe the fuzzy random distribution of $\stackrel{\sim }{\upomega }$ as follows:

4 $$\tilde{\omega }k = \left\{ {\begin{array}{*{20}c} {\begin{array}{*{20}c} {0 k < 0.4} \\ {10 \times \left( {k - 0.4} \right) 0.4 \le k \le 0.5} \\ {2.5 \times \left( {0.9 - k} \right) 0.5 \le k \le 0.9} \\ \end{array} } \\ {1 k > 0.9} \\ \end{array} } \right.$$

where k represents the current number of iterations.

(2) In the algorithm iteration process, there is fuzzy randomness at each flight position of the particles. Considering that there is also uncertainty in practical engineering problems, the fuzzy random coefficient $\tilde{\lambda }_{{(k}}$ was introduced into Eq. (5) to balance fuzziness of the flight path and convergence.

5 $$x_{i,d}^{k + 1} = \tilde{\lambda }_{{(k}} x_{i,d}^{k} + v_{i,d}^{k + 1}$$

Here, $\tilde{\lambda }_{{(k}}$ represents the fuzzy random coefficient, and K represents the current number of iterations.

According to the fuzzy interval values of c_1_ and c_2_, triangular membership function (intermediate fuzzy random distribution) was also used to describe the fuzzy random distribution of ${\tilde{\lambda }}$ as follows:

6 $$\mu_{{\tilde{\lambda }}} = \left\{ {\begin{array}{*{20}c} {0} & {\lambda < c_{1} } \\ {2\frac{{\lambda - c_{1} }}{{c_{2} - c_{1} }},} & {c_{1} \le \lambda \le \frac{{c_{1} + c_{2} }}{2}} \\ {2\frac{{c_{2} - \lambda }}{{c_{2} - c_{1} }},} & {\frac{{c_{1} + c_{2} }}{2} \le \lambda \le c_{2} } \\ {1,} & {\lambda > c_{2} } \\ \end{array} } \right.$$

**Figure 1:**
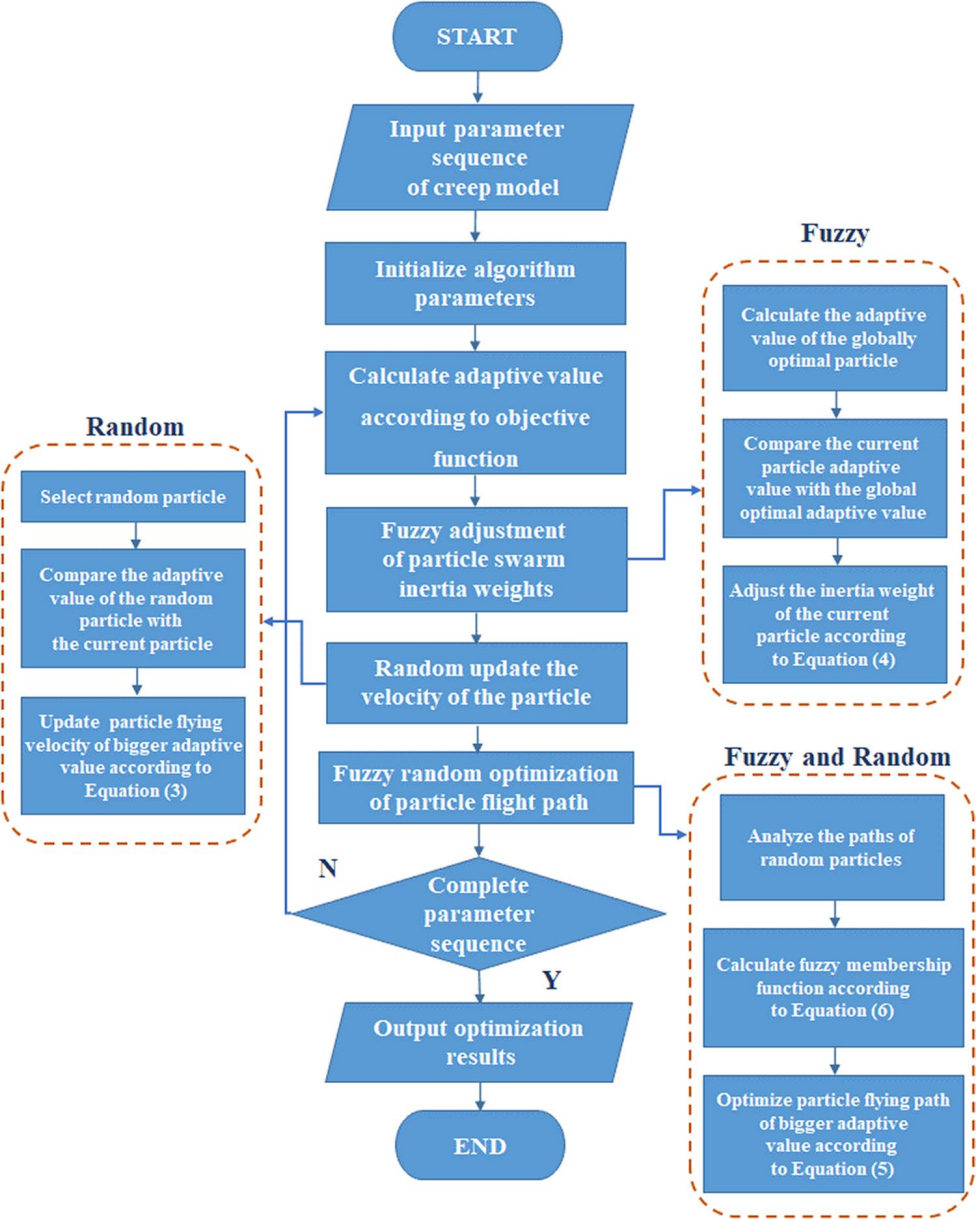
Flowchart of the fuzzy random particle swarm optimization algorithm.

After the foregoing improvements, the fuzzy random particle swarm algorithm can improve the flying speed of particles and accelerate the convergence^34–36^. Moreover, it can adapt well to the fuzzy random mechanical properties of the actual working conditions in underground geotechnical engineering and can be an effective tool for intelligent optimization of related parameters and models. The optimization process of creep model parameters by using the improved fuzzy random particle swarm algorithm is shown in Fig. 1.

Thus, the improved fuzzy random particle swarm optimization algorithm has the following characteristics:

1. When dealing with high-dimensional complex problems, the improved algorithm has fast search speed and high convergence efficiency.
2. The fuzzy random particle swarm optimization algorithm considers both fuzziness and randomness, and has high optimization accuracy, which can better analyze the uncertain problems in practical engineering.
3. For the optimization of discrete value problem, the advantages of the new algorithm are not obvious, and it is easy to fall into local optimal

## Fuzzy random optimization of Burgers creep damage model

### Burgers creep model

**Figure 2:**
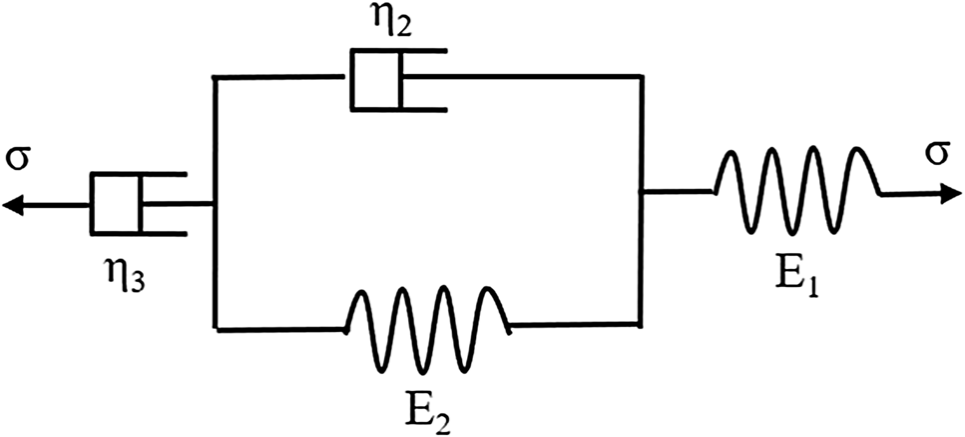
Original Burgers element model.

There are two main creep models of frozen soil: the empirical model and the element model. The element creep model is composed of different combinations of springs, clay pots, friction plates, and other elements^37,38^. For example, the Burgers creep model is composed of Maxwell and Kelvin bodies connected in series. The model elements and creep equation are presented in Fig. 2 and Eq. (7), respectively^39–41^.

7 $$\varepsilon = \frac{\sigma }{{E_{1} }} + \frac{\sigma }{{E_{2} }}\left[ {1 - e^{{\left( { - \frac{{E_{2} }}{{\eta_{2} }}t} \right)}} } \right] + \frac{\sigma }{{\eta_{3} }}t$$

Here, t represents the creep time, $\varepsilon$ represents the axial strain, $\sigma$ represents the constant stress in the test, and ${E}_{1},{E}_{2},{\eta }_{2}, \mathrm{and} {\eta }_{3}$ are the creep parameters of the Burgers model.

### Improved Burgers creep damage model

Numerous creep experiments revealed that the creep process of frozen soil involves a stable creep stage (σ $\le$ σ_s_) and an accelerated creep stage (σ > σ_s_), where σ_s_ represents the yield stress of the frozen soil. The original Burgers model can accurately describe the characteristics of the stable creep stage. However, the accelerated creep stage cannot be accurately characterized^42,43^. To describe the whole process of frozen soil creep with the improved model accurately, it is necessary to establish an elastic damage element model D according to the damage mechanics theory for simulating the deformation process in the accelerated creep stage. Then, the elastic damage element D is combined with the original Burgers model in series to establish the Burgers damage creep model.

**Figure 3:**
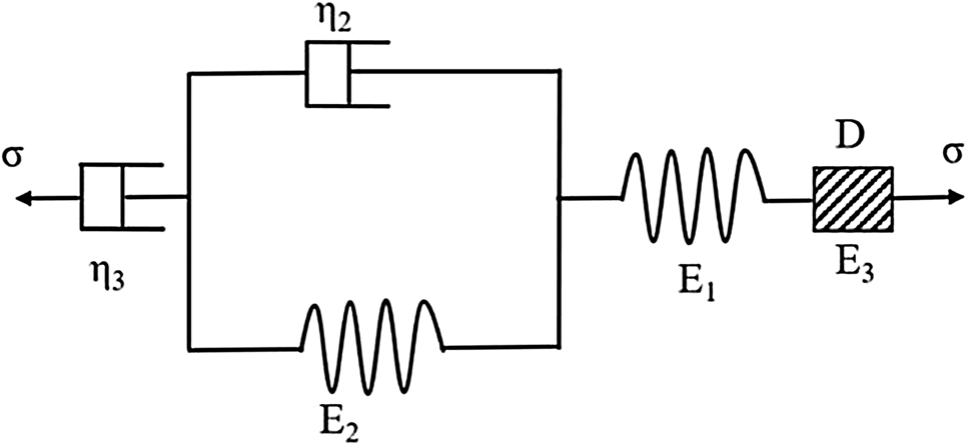
Burgers damage creep model of the elastic damage element combined in series.

When the frozen soil enters the stage of accelerated creep (t* > *t*), the creep rate increases continuously. Consequently, the damage inside the frozen soil accumulates continuously, and the soil is finally destroyed. Therefore, a nonlinear time-varying elastic element^44,45^ is added to the original Burgers model to describe the damage in the accelerated creep stage, as shown in Fig. 3.

According to the preliminary creep test, the creep curve in the accelerated creep stage is close to the exponential function. We can assume that the evolution of the creep damage in the accelerated creep process is given by an exponential function between the stress level and time. Then, the evolution Eq. ^46^ for the damage element D in the accelerated creep stage of frozen soil can be expressed as follows:

8 $$D(t = 1 - \left( {R_{1} \sigma } \right)^{{ - R_{2} \left( {t - t^{*} } \right)}}$$

where t* represents the time after the frozen soil enters the accelerated creep stage, and R1 and R2 are damage parameters of frozen-soil materials. As indicated by the formula, when *t* = t*, D = 0, indicating the nondestructive state of the frozen soil. When *t* approaches infinity, D = 1, indicating that the frozen soil is completely destroyed and loses its bearing capacity.

Additionally, the elastic modulus can be derived according to the assumption of equivalent strain, and the time-dependent damage^47,48^ variable can be defined as follows:

9 $$D(t = 1 - \frac{{E_{0} \left( t \right)}}{{E_{3} }}$$

where E_0_ represents the Young’s modulus of a lossy material, and E_3_ represents the elastic modulus of a nondestructive material.

The stress–strain relationship of an elastic element can be determined using Hooke’s law.

10 $$\varepsilon = \frac{\sigma }{{E_{3} }}$$

In accordance with the strain equivalence principle of damage mechanics, by substituting the Young’s modulus E_0_ of a lossy material in Eq. (9) for the elastic modulus E_3_ of a lossless material in Eq. (10), the time-dependent damage constitutive model of an elastic element can be obtained.

11 $$\varepsilon = \frac{\sigma }{{E_{3} \left( {1 - D\left( t \right)} \right)}}$$

By substituting the damage element evolution equation into the damage constitutive model, the creep damage equation for the accelerated creep stage of frozen soil can be derived.

12 $$\varepsilon^{*} = \frac{\sigma }{{E_{3} }}\left( {R_{1} \sigma } \right)^{{R_{2} \left( {t - t^{*} } \right)}}$$

Here, ε* represents the damage strain of frozen soil in the accelerated creep stage.

Finally, according to the superposition principle, the Burgers creep damage model consists of a nonlinear time-dependent elastic damage element combined with the original Burgers model in series. The improved creep damage model is expressed as follows:

13 $$\varepsilon = \frac{\sigma }{{E_{1} }} + \frac{\sigma }{{E_{2} }}\left[ {1 - e^{{\left( { - \frac{{E_{2} }}{{\eta_{2} }}t} \right)}} } \right] + \frac{\sigma }{{\eta_{3} }}t + \frac{\sigma }{{E_{3} }}\left( {R_{1} \sigma } \right)^{{R_{2} \left( {t - t^{*} } \right)}}$$

where $E_{1} ,E_{2} ,E_{3} ,\eta_{2} , {\text{and}} \eta_{3}$ are the creep parameters of the Burgers creep damage model.

Based on the Burgers creep damage model, the original creep parameters were inversed by using the traditional particle swarm algorithm. The original parameter data are shown in Tables 1 and 2.

**Table 1:** Original parameters of the Burgers creep damage model (σ $\le$ σ_s_).

Temperature (°C)	Original Burgers creep damage model parameters			
	$E_{1 }$(MPa)	$E_{2}$(MPa)	$\eta_{2}$(MPa·h)	$\eta_{3}$(MPa·h)
− 10	175.26	158.13	267.34	230.61
− 15	132.04	126.72	305.77	149.25
− 20	86.37	104.99	280.15	176.08
− 25	119.23	73.06	195.30	96.37

**Table 2:** Original parameters of the Burgers creep damage model (σ > σ_s_).

Temperature (°C)	Original Burgers creep damage model parameters						
	${E}_{1} ($MPa)	${E}_{2} ($MPa)	${E}_{3} ($MPa)	${\eta }_{2} ($MPa·h)	${\eta }_{3} ($MPa·h)	${R}_{1}$	${R}_{2}$
− 10	316.75	257.81	240.26	468.03	349.21	0.218	0.357
− 15	268.04	184.30	199.72	409.74	176.59	0.432	0.418
− 20	159.32	216.79	158.35	334.12	202.86	0.397	0.653
− 25	210.67	156.46	191.58	352.69	143.07	0.516	0.592

### Fuzzy random optimization of Burgers creep damage model parameters

Based on the model tests and engineering data, the improved fuzzy random particle swarm optimization algorithm^49–51^ was simulated using MATLAB (a numerical calculation software) to conduct a fuzzy random optimization of the creep parameters of the improved Burgers damage model. The parameters of the fuzzy random particle swarm algorithm were initialized as follows: a maximum of 600 iteration steps, a calculation error of 10^–2^, a maximum acceleration of 20, a particle population of m = 150, and acceleration constants of c_1_ = 2.8 and c_2_ = 1.3. The inertia weight was initially $\stackrel{\sim }{\omega }$(0) = 0.9 and then took a fuzzy value in the interval of [0.4, 0.9] according to Eq. (4), and the fuzzy random coefficient was initially 2 and then took a fuzzy value in the interval of [1.3, 2.8] according to Eq. (5). To distinguish the stable creep stage from the unstable creep stage, the global optimal values of the Burger creep damage model parameters were obtained through the iteration of the fuzzy random particle swarm optimization algorithm. The results are presented in Tables 3 and 4.

**Table 3:** Fuzzy random optimization of the Burgers creep damage model parameters (σ $\le$ σ_s_).

Temperature (°C)	Optimization results for creep parameters			
	${E}_{1}$(MPa)	${E}_{2}$(MPa)	${\eta }_{2}$(MPa·h)	${\eta }_{3}$(MPa·h)
− 10	216.07	136.20	364.25	251.93
− 15	154.98	117.32	337.09	163.47
− 20	129.53	96.14	320.56	109.25
− 25	103.26	83.59	284.30	79.61

**Table 4:** Fuzzy random optimization of the Burgers creep damage model parameters (σ > σ_s_).

Temperature (°C)	Optimization results for creep parameters						
	${E}_{1} ($MPa)	${E}_{2} ($MPa)	${E}_{3} ($MPa)	${\eta }_{2} ($MPa·h)	${\eta }_{3} ($MPa·h)	${R}_{1}$	${R}_{2}$
− 10	376.35	265.42	235.18	508.65	317.09	0.167	0.295
− 15	281.04	219.65	204.92	437.20	152.34	0.258	0.384
− 20	235. 92	158.23	176.35	362.59	114.26	0.391	0.560
− 25	213.68	94.77	152.46	309.12	93.85	0.514	0.672

The obtained values of the creep parameters were substituted into Eq. (13) for different cases to obtain the improved Burgers creep damage model equation.

### Engineering example verification

**Figure 4:**
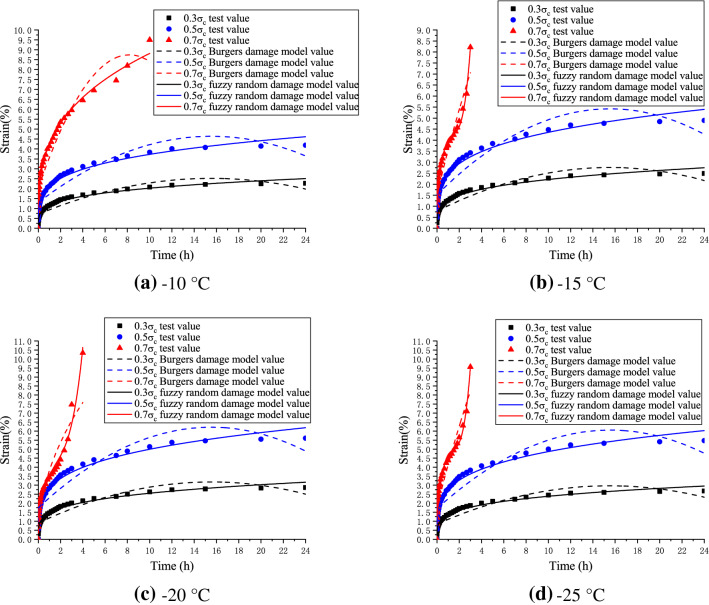
Comparison of original Burgers damage creep values, fuzzy random damage model values with experimental values.

To verify the applicability of the optimized parameters and improved model to the creep of frozen silty clay, the silty clay in the freezing engineering of the North Street Station of Nantong metro line 2 was selected as a soil sample. Under different temperatures and stresses, the creep test values of frozen soil samples, the original Burgers damage creep model values, and the Burgers creep damage model values after fuzzy random optimization were compared. The results are shown in Fig. 4.

**Figure 5:**
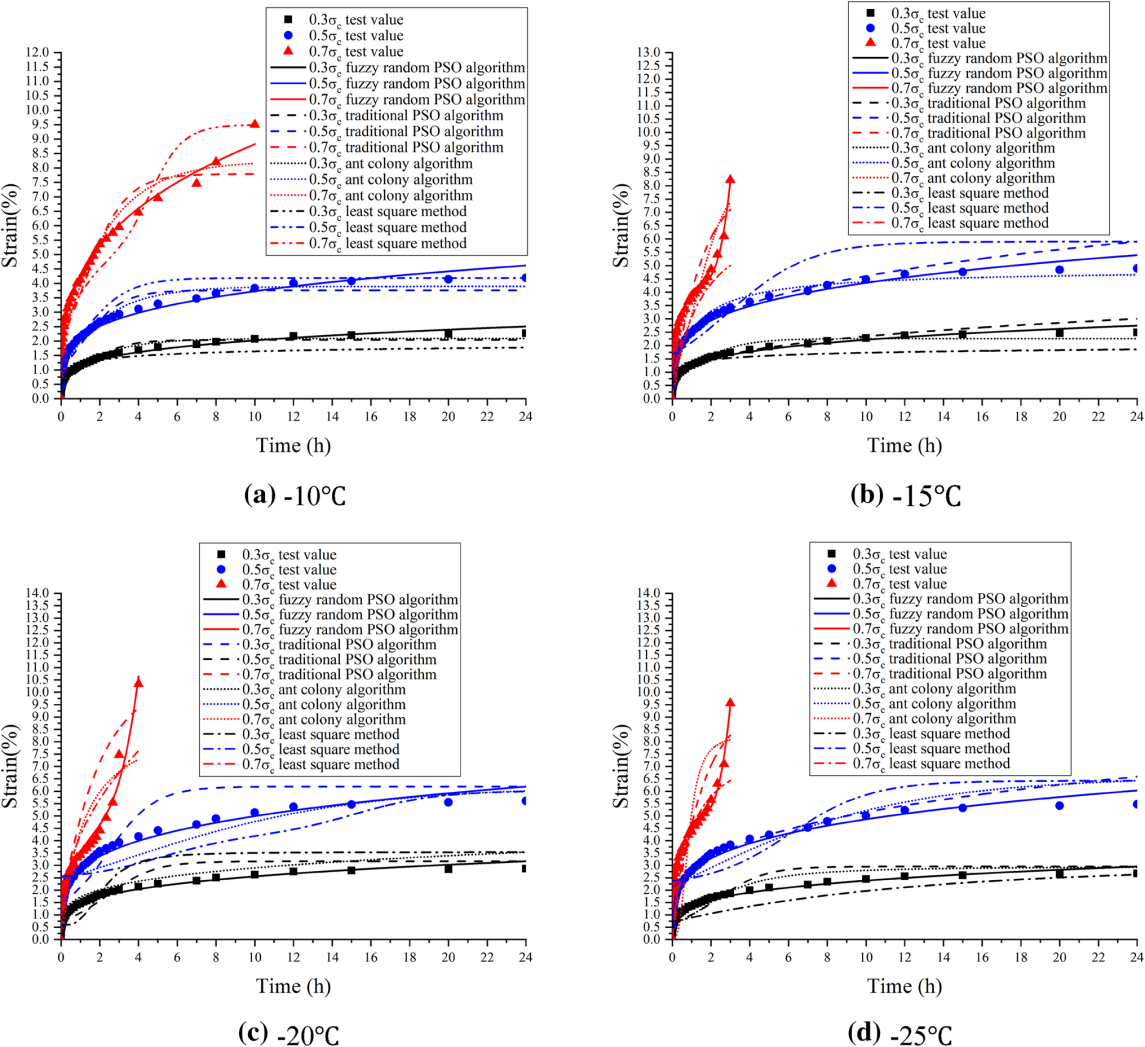
Comparison of least square method, ant colony algorithm, traditional particle swarm algorithm, fuzzy random particle swarm algorithm with experimental values.

In order to verify the superiority of fuzzy random particle swarm optimization, different algorithms were used to optimize Burgers creep damage model under different temperature and stress conditions. The creep model optimization values of least square method, ant colony algorithm, traditional particle swarm algorithm and fuzzy random particle swarm algorithm were compared with the test values of frozen soil samples. The results are shown in Fig. 5.

**Figure 6:**
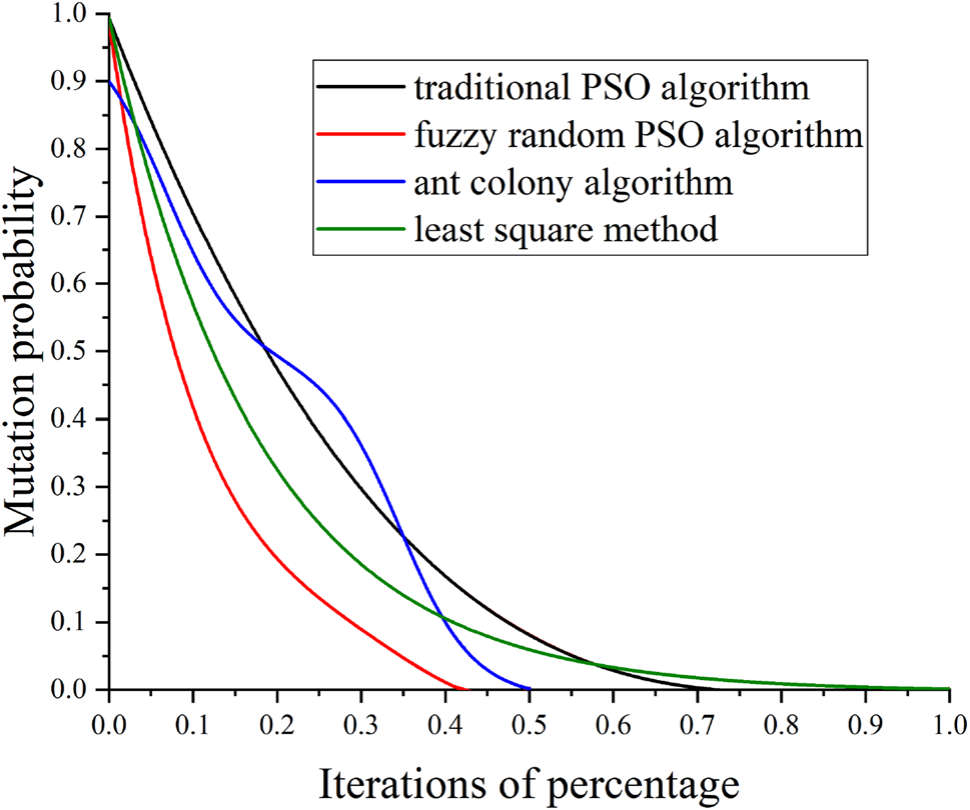
Efficiency comparison of different algorithms.

The engineering example (Fig. 4) revealed that the Burgers creep damage model optimized by the fuzzy random particle swarm algorithm can better fit the experimental values of frozen soil creep at different temperatures and pressures than the original one, for both the stable creep stage and the accelerated creep stage. The reliability of the new creep model is improved by 5–10%. Therefore, the optimized Burgers creep damage model accurately characterized the whole creep stages of frozen silty clay in Nantong metro construction. According to Fig. 5, the improved fuzzy random particle swarm algorithm has higher optimization accuracy than conventional algorithms such as traditional particle swarm algorithm, ant colony algorithm and least square method. Experiments show that the optimization accuracy of the new algorithm is increased by 15–25%, and it is easier to find the global optimal solution, indicating that its engineering applicability is wider. In addition, with the increase of problem scale, the convergence efficiency of fuzzy random particle swarm optimization algorithm is about 10–20% higher than that of conventional algorithms, as shown in Fig. 6.

## Conclusions

Uniaxial tests of the frozen silty clay in the frozen construction layer of Nantong metro were performed to determine the compressive and creep characteristics. Based on previous studies, the traditional particle swarm algorithm was improved, and a Burgers creep damage model was established and optimized for the fuzzy randomness of underground geotechnical engineering. The following conclusions are drawn.

1. Under the condition of uniaxial compression, the compressive strength of frozen silty clay has an inversely proportional relationship with the temperature. Generally, the uniaxial compressive strength increases with a decrease in the temperature. The ultimate damage deformation of the soil specimen was between 7 and 15%, indicating shear failure. Owing to the complex effects of the temperature and ground pressure, the mechanical properties of underground frozen silty clay have significant uncertainty.
2. The creep process of frozen silty clay is significantly affected by the freezing temperature and stress level, which is accompanied by fuzzy randomness. In general, the creep value increases with an increase in the stress. Under a low or moderate stress, the creep of frozen soil is stable. When the stress is high, the creep tends to accelerate.
3. An improved fuzzy random particle swarm algorithm was obtained wherein the inertia weight and fuzzy random coefficient are used to perform a fuzzy random correction of the flight speed and position in the iteration process. The improved particle swarm algorithm has a lower mutation probability than the traditional algorithm and can more easily find the global optimal solution of multidimensional complex problems accompanied by fuzzy randomness. Moreover, the new algorithm has a convergence efficiency 10–20% and a optimization accuracy 15–25% higher than conventional algorithms, and its engineering applicability is wider.
4. According to the theory of damage mechanics, an elastic damage element model was used to simulate the accelerated creep stage, and then the elastic damage element was combined in series with the original Burgers model to establish a Burgers creep damage model. On this basis, the improved fuzzy random particle swarm algorithm was used to optimize the creep parameters of the Burgers damage model at different temperatures and pressure levels. Finally, an engineering example revealed that the reliability of the optimized creep parameters and damage model is improved by 5–10%, which can describe the whole creep process of underground geotechnical engineering more accurately than the original one.

## Supplementary Information

Supplementary Information.

## Data Availability

All data, models, and code generated or used during the study appear in the submitted article.
