# Supplementary material for: Optimization of Burgers creep damage model of frozen silty clay based on fuzzy random particle swarm algorithm
Source: Sci Rep. 2021 Sep 23;11:18974. doi: 10.1038/s41598-021-98374-1 (PMC8460797; doi:10.1038/s41598-021-98374-1)
Supplement: Supplementary file 1 — Supplementary Information. [file 41598_2021_98374_MOESM1_ESM.docx]

Supplementary Appendix

A uniaxial test of the frozen silty clay was performed using a cryogenic frozen rock and soil test system (model: ZTCR-2000). As shown in Fig. 1, the maximum loading capacity of the test system was 2000 kN, and the force measurement precision was 1%. When the freezing test was performed, the lowest temperature of the system was –40 ℃, the temperature control precision was 0.1 ℃, and the deformation measurement accuracy was 1%. Throughout the test process, the stress loading and data acquisition were automatically performed by the computer according to the set parameters.


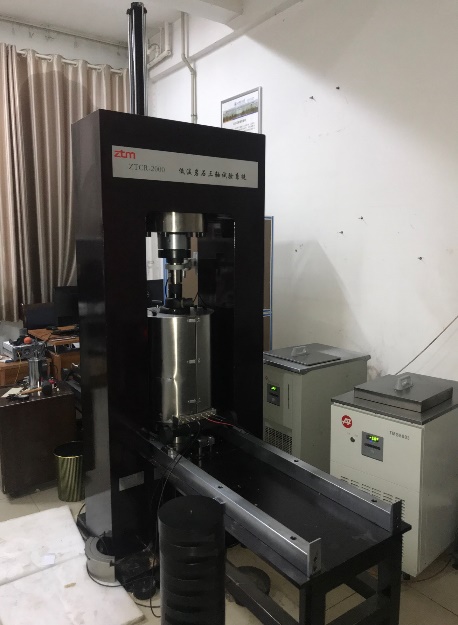


(a) Test host


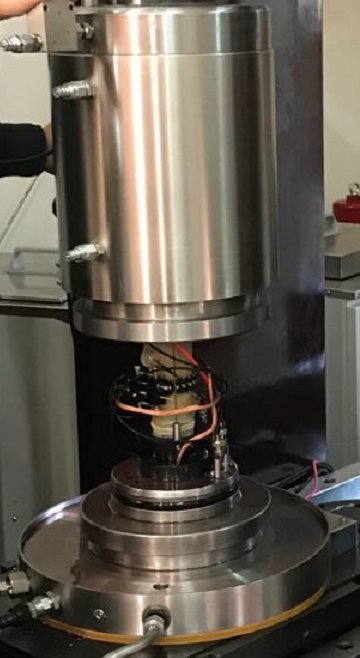


(b) Install the specimen

Fig. 1. ZTCR-2000 cryogenic frozen rock and soil test system.

The uniaxial compression experiment was performed according to the foregoing steps, with three specimens for each temperature condition. Taking a group of soil samples collected from the freezing engineering of the contact channel of Nantong University station of metro line 1 as an example, the results of the uniaxial compression experiment are presented in Table 1.

Table 1

Uniaxial compression test of silty clay.

| Temperature (℃) | -10 | -15 | -20 | -25 |
| --- | --- | --- | --- | --- |
| Compressive strength (MPa) | 3.91 | 5.05 | 4.48 | 8.59 |
|  | 4.84 | 4.10 | 5.07 | 9.21 |
|  | 4.06 | 4.85 | 7.11 | 7.91 |
| Average value (MPa) | 4.27 | 4.67 | 5.55 | 8.57 |

The results indicated that under uniaxial compression, the compressive strength of the frozen silty clay specimens had an inversely proportional relationship with the temperature. Generally, the uniaxial compressive strength increased with a decrease in the temperature of the specimen. Additionally, the mechanical properties of the frozen silty clay were uncertain under the effects of the temperature, water content, and ground pressure.

In the uniaxial compression experiment, two instruments for measuring the displacement were arranged symmetrically in the corresponding direction of the specimen. The relationship between the axial deformation (strain) and the load (stress) of the specimen at different temperatures was established, as shown in Fig. 2.

(a) -10 ℃ (b) -15 ℃

(c) -20 ℃ (d) -25 ℃

Fig. 2. Axial stress–strain relationship at different temperatures.

The relationship between the lateral deformation (strain) and the load (stress) of the specimen at different temperatures was established, as shown in Fig. 3.

(a) -10 ℃ (b) -15 ℃

(c) -20 ℃

(d) -25 ℃

Fig. 3. Lateral stress–strain relationship at different temperatures.

According to the stress-strain curves of the frozen silty clay specimens, even if the stress-strain relationship of different specimens are accompanied by uncertainty at the same temperature. Generally, the curves gradually changed from a hardening tendency to a softening tendency, and the final damage deformation of the specimen was between 7% and 15%. The frozen silty clay specimens damaged in the test mainly underwent shear failure (Fig. 4). The relevant mechanism should be fully considered in underground freezing engineering.

**
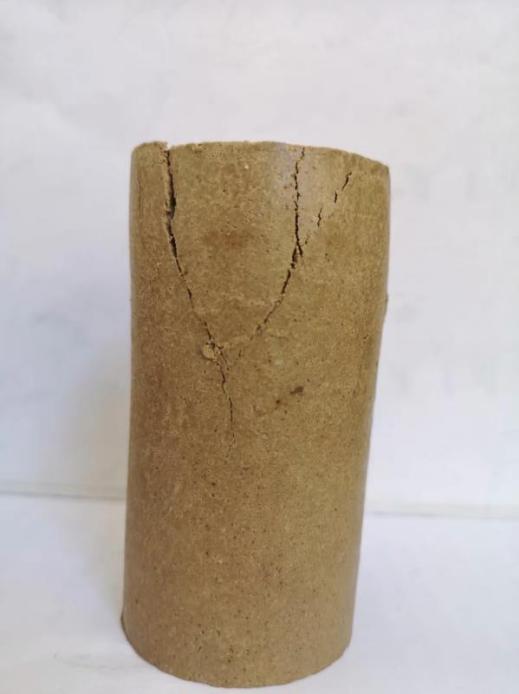
**

Fig. 4. Damaged specimen of frozen silty clay.

To investigate the creep characteristics of the frozen soil, uniaxial creep tests with stress levels of 0.3$\sigma_{c}$ , 0.5$\sigma_{c}$, and 0.7$\sigma_{c}$ were performed on silty clay specimens by using the multi-specimen method at –10, –15, –20, and –25 ℃. $\sigma_{c}$ represents the uniaxial compressive strength, which was evaluated according to Table 1. The final strain and creep time for each silty clay specimen of different depths are presented in Table 2.

Table 2

Uniaxial creep tests of frozen silty clay.

(a) Group 1 specimens (depth of 16.7 m)

| Temperature (℃) | Stress (MPa) | Strain (%) | Time (h) |
| --- | --- | --- | --- |
| -10 | 1.07 | 1.86 | 24 |
|  | 1.79 | 3.55 | 24 |
|  | 2.51 | 9.54 | 4 |
| -15 | 1.36 | 1.92 | 24 |
|  | 2.27 | 3.94 | 24 |
|  | 3.17 | 9.16 | 7 |
| -20 | 1.65 | 1.76 | 24 |
|  | 2.76 | 3.64 | 24 |
|  | 3.86 | 8.87 | 4 |
| -25 | 1.98 | 1.78 | 24 |
|  | 3.31 | 3.62 | 24 |
|  | 4.63 | 8.96 | 5 |

(b) Group 2 specimens (depth of 18.4 m)

| Temperature (℃) | Stress (MPa) | Strain (%) | Time (h) |
| --- | --- | --- | --- |
| -10 | 0.81 | 2.28 | 24 |
|  | 1.35 | 4.39 | 24 |
|  | 1.89 | 7.57 | 4 |
| -15 | 1.02 | 2.34 | 24 |
|  | 1.69 | 4.45 | 24 |
|  | 2.37 | 7.76 | 5 |
| -20 | 1.45 | 2.45 | 24 |
|  | 2.41 | 4.79 | 24 |
|  | 3.38 | 7.68 | 4 |
| -25 | 2.47 | 2.52 | 24 |
|  | 4.13 | 5.01 | 24 |
|  | 5.78 | 8.25 | 5 |

(c) Group 3 specimens (depth of 21.5 m)

| Temperature (℃) | Stress (MPa) | Strain (%) | Time (h) |
| --- | --- | --- | --- |
| -10 | 0.71 | 1.98 | 24 |
|  | 1.19 | 3.69 | 24 |
|  | 1.67 | 9.32 | 7 |
| -15 | 1.01 | 1.71 | 24 |
|  | 1.68 | 3.57 | 24 |
|  | 2.35 | 9.40 | 5 |
| -20 | 1.37 | 1.65 | 24 |
|  | 2.26 | 3.91 | 24 |
|  | 3.16 | 12.4 | 7 |
| -25 | 1.98 | 1.87 | 24 |
|  | 3.29 | 4.03 | 24 |
|  | 4.61 | 10.50 | 5 |

Through the uniaxial creep tests under different temperature and stress conditions, the creep relationship curve for the frozen silty clay was obtained, as shown in Fig. 5~8.

 (a)-10 ℃ (c) -20 ℃

 (b) -15 ℃

(d) -25 ℃

Fig. 5. Creep curves for silty clay at different temperatures (depth of 16.7 m).

(a) -10 ℃

(b) -15 ℃

(c) -20 ℃

(d) -25 ℃

Fig. 6. Creep curves for silty clay at different temperatures (depth of 18.4 m).

(a) -10 ℃

 (c) -20 ℃

 (b) -15 ℃

(d) -25 ℃

Fig. 7. Creep curves for silty clay at different temperatures (depth of 21.5 m).

(a) 0.3σ_c_

(b) 0.5σ_c_

(c) 0.7σ_c_

Fig. 8. Creep curves for silty clay under different stress levels.
